# Spinocerebellar ataxia type 28 in a Chinese pedigree: A case report and literature review

**DOI:** 10.1097/MD.0000000000028008

**Published:** 2021-12-17

**Authors:** Xiaoyang Liu, Linlin Wang, Jiajun Chen, Chunyang Kang, Jia Li

**Affiliations:** Department of Neurology, China–Japan Union Hospital of Jilin University, No 126, Xiantai Street, Changchun, Jilin, China.

**Keywords:** gene mutation, pedigree, spinocerebellar ataxia

## Abstract

**Rationale:**

Spinocerebellar ataxia (SCA) is a common neurogenetic disease that mainly manifests as ataxia of posture, gait, and limbs, cerebellar dysarthria, and cerebellar and supranuclear eye movement disorders. SCA has been found to include many subtypes, which are mainly mapped to 2 genetic patterns: autosomal dominant cerebellar ataxia and autosomal recessive cerebellar ataxia. Molecular genetic diagnosis functions as a necessity in its clinical diagnosis and treatment. In preliminary clinical work, we identified a family of SCA28 with rare gene mutation.

**Patient concerns:**

There are 5 patients in this family. The proband is a 32 year-old male, he mainly manifest unsteady steps for more than 7 months. The daughter of his younger maternal uncle gradually had unsteady steps and unclear speech for 5 years. The proband's mother, uncle and grandfather had similar symptoms, but they all died.

**Diagnosis:**

After Brain magnetic resonance imaging, whole exome sequencing and Sanger validation, the patients presented a c.1852A > G missense mutation in the exon region of AFG3L2 gene. The other family members revealed no AFG3L2 mutations. SCA28 is the one uniquely caused by a pathogenic variation in the mitochondrial protein AFG3L2. Combined with the clinical manifestations, auxiliary examinations and sequencing results of the patients (III-3 and III-5), the diagnosis of SCA28 was suspected.

**Interventions:**

The patients did not receive any drug treatment and the proband receive rehabilitation treatment.

**Outcomes:**

The symptoms of ataxia were still progressively aggravated.

**Lessons:**

Molecular genetic diagnosis is necessary for ataxia. We here report the case and review the literature.

## 1 Introduction

Spinocerebellar ataxia (SCA) is a common neurogenetic disease that mainly manifests as ataxia of posture, gait, and limbs, cerebellar dysarthria, and cerebellar and supranuclear eye movement disorders, and is a highly genetically and clinically heterogeneous disease that accounts for approximately 10% to 15% of nervous system genetic disorders. SCA is a progressive neurodegenerative disease that includes many subtypes, which are mainly mapped to 2 genetic patterns: autosomal dominant cerebellar ataxia and autosomal recessive cerebellar ataxia. Molecular genetic diagnosis is necessary for clinical diagnosis and treatment. The clinical manifestations of the different HA subtypes are similar, making a simple diagnosis difficult. Thus, genetic testing has proven to be very valuable for patients with HA. Genetic testing techniques are currently the most efficient tools for HA diagnosis and classification, and in our previous clinical work, we found a family with ataxia who presented a c.1852A > G missense mutation in the exon region of the AFG3L2 gene by whole exome sequencing and Sanger validation. SCA28 was suspected, which has not been reported in previous literature. Here, we report a case and review the literature.

## 2 Case presentation

The proband (III-3) (32 year-old, male) visited the Department of Neurology of our hospital for “unsteady steps for more than 7 months” More than 7 months prior to admission, the patient gradually had unsteady steps with no obvious predisposing causes, manifested as walking with a rolling gait. The patient's condition gradually worsened and manifested with increased step distance and drunk walking from side to side at admission. The patient complained of recent difficulties in eye opening, involuntary tremor of both upper limbs, and catatonia, which were aggravated after activities.

Neurological examination: Conscious mind, dysarthria, normal memory, calculation and orientation power, normal visual acuity and field of both eyes, free movement of both eyes in all directions, horizontal nystagmus when both eyes look to the left, ptosis of both eyes, unstable and inaccurate bilateral finger-nose test, clumsy bilateral alternating bilateral movements, bilateral heel-knee-shin test, and no bilateral pathological reflex.

**Figure 1:**
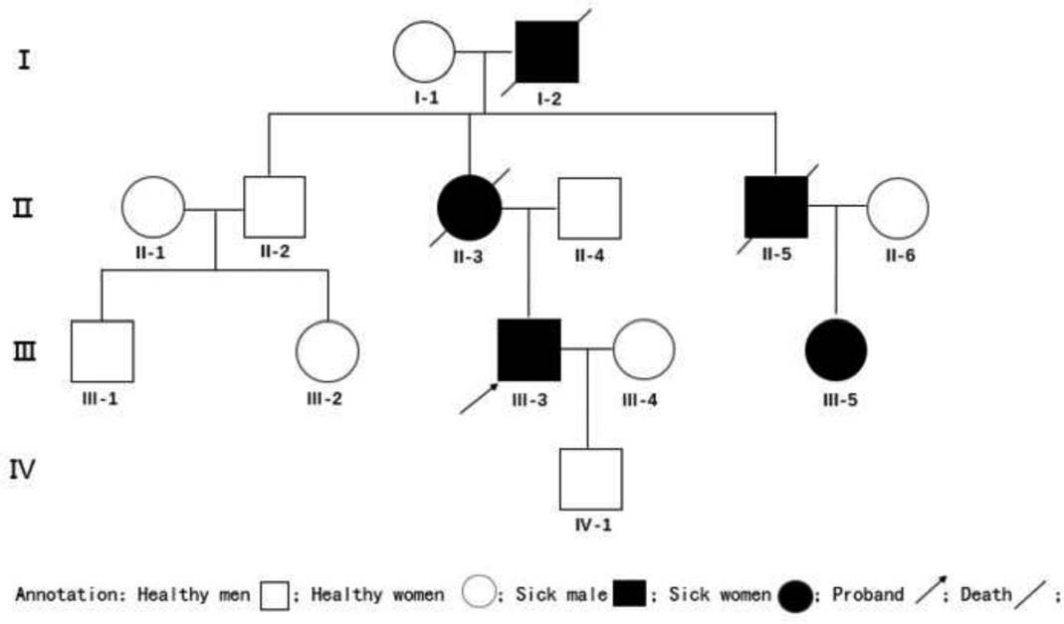
Head MRI of the proband (III-3) showed cerebellar atrophy with no obvious abnormal signals in the rest. Cervical MRI showed no atrophy or thinning in cervical cord, and no obvious abnormal signal was found. MRI = magnetic resonance imaging.

Auxiliary examinations: Head magnetic resonance imaging (MRI) of the proband (III-3) showed cerebellar atrophy with no clear abnormal signals. Cervical MRI suggested no atrophy or thinning of the cervical cord (Fig. 1).

**Figure 2:**
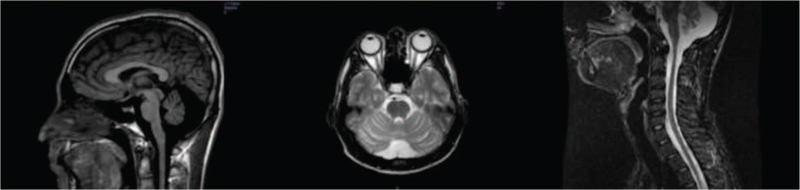
Pedigree Map.

Family surveys: Figure 2 shows the family history of the proband (III-3). The mother (II-3) developed similar symptoms in her 30s, was paralyzed in bed in the following days and died in her 40s. The elderly maternal uncle (II-2) and his children (III-1, III-2) have no clear clinical manifestations. The younger maternal uncle (II-5) began to suffer from illness in his 20s and died from disease in his 30s.

**Figure 3:**
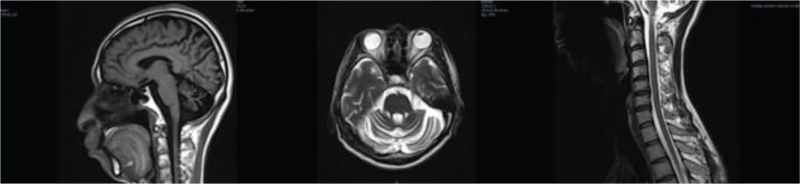
Head MRI of the patient III-5 showed cerebellar atrophy with no obvious abnormal signals in the rest. Cervical MRI showed no atrophy or thinning in cervical cord, and no obvious abnormal signal was presented. MRI = magnetic resonance imaging.

The daughter of the younger maternal uncle (III-5) (25-year-old now) gradually had unsteady steps and unclear speech with no obvious incentive at around 20 years old, manifested as a slightly wide step base, unstraight walking, influent speech, and appearance of choking cough in drinking water in the past year (Table 1). Neurological examination: conscious mind, dysarthria, horizontal nystagmus when both eyes look to the left and right, active tendon reflex of limbs, unstable and inaccurate bilateral finger-nose test, clumsy bilateral alternating movements, and bilateral heel-knee-shin test. Auxiliary examination: Head magnetic resonance imaging suggested cerebellar atrophy (Fig. 3).

**Table 1:** Clinical manifestations of 2 patients in a SCA28 family.

No.	Onset age	Ataxia	Dysarthria	Nystagmus	Choking and cough in drinking water	Pyramidal tract signs	Tendon reflex	Muscle atrophy	Ptosis	Tremor
III-3	32-yr-old	+	+	Horizontal nystagmus	+	–	Normal	–	+	+
III-5	25-yr-old	+	+	Horizontal nystagmus	+	–	Active	_	_	_

**Figure 4:**
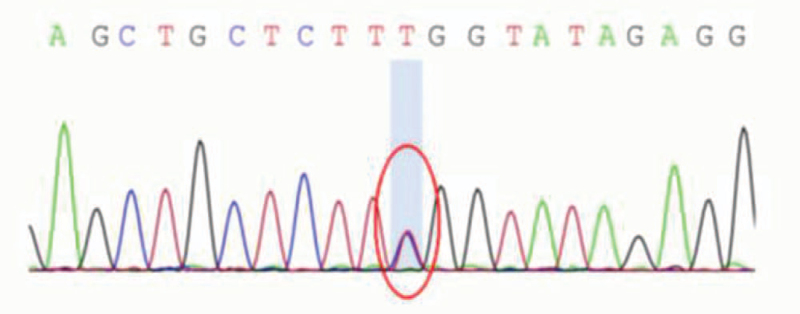
Proband (III-3) gene sequencing.

**Figure 5:**
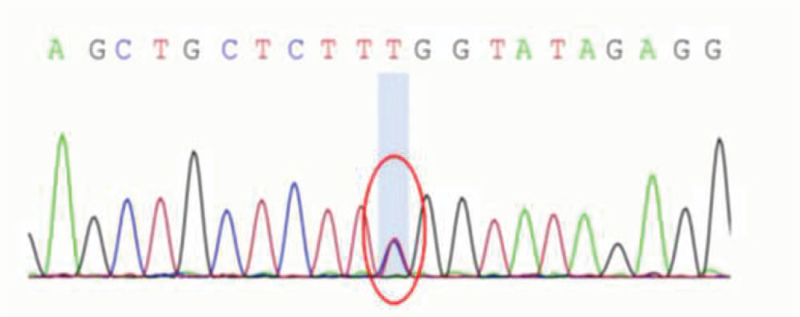
Patient III-5 gene sequencing.

After obtaining informed consent from patients and their families, serum samples of the proband (III-3) and his cousin (III-5) were collected to identify genes involved in the pathogenesis of SCA. The blood samples were sent to the Beijing High Trust Diagnostic for Whole Exome Sequencing. Sanger sequencing of the patients (III-3 and III-5) revealed a c.1852A > G missense mutation in the exon region of the AFG3L2 gene, resulting in a lysine-glutamate amino acid substitution (p.K618E) (Figs. 4 and 5). Sanger sequencing of the other family members (II-1, III-1) revealed no AFG3L2 mutations. We checked the human gene mutation database and found that the gene mutations reported here had not been reported before. According to ACGS recommended guidelines, we thought this mutation considered pathogenic. SCA28 is the one uniquely caused by a pathogenic variation in the mitochondrial protein AFG3L2. Combined with the clinical manifestations, auxiliary examinations and sequencing results of the patients (III-3 and III-5), the diagnosis of SCA28 was suspected. The patients did not receive any drug treatment and the proband receive rehabilitation treatment. The symptoms of ataxia were still progressively aggravated.

## 3 Discussion

SCA accounts for 10% to 15% of the neurogenetic diseases with a prevalence rate of (1-4) /100,000 across all ethnic groups, whereas the prevalence rate varies significantly in different geographical regions and ethnic groups.^[1]^ Based on the order of discovered pathogenic genes, more than 40 types of SCA can be divided, including 46 loci/genes discovered and 35 causal genes identified.^[2]^ The genetic diversity of SCA falls in trinucleotide repeat expansions (SCA1, 2, 3, 6, 7, 8, 12, 17, dentatorubral-pallidoluysian atrophy), pentanucleotide repeat expansions (SCA10 and 31), hexanucleotide repeat expansions (SCA36), conventional mutations (SCA5, 11, 13, 14, 15/16, 18, 19/22, 21, 23, 26, 27, 28, 29, 34, 35, 38, and 40), and types of unidentified responsible genes (SCA4, 20, 25, 30, 32, and 37).^[3]^ Of the current autosomal dominant SCA subtypes, only a small number of pathogenic genes have been identified. As reported, 20% to 40% of SCA families have no gene mutations.^[4]^ Varying functions of disease-associated genes cause complex heterogeneity in the pathogenesis of cerebellar degeneration and ataxia.

AFG3L2 (ATPase family gene 3-like 2) belongs to the AAA protease subfamily (ATPase associated with various cellular activities) and is involved in the assembly of m-AAA proteases in the inner mitochondrial membrane (IMM), which is a hetero-oligomers.^[5^] m-AAA proteases are key components of the quality control system of IMM that can mediate the selective degradation of unassembled and damaged proteins.^[6]^ In addition, m-AAA proteases are equipped with other functions, such as promoting mitochondrial protein synthesis,^[7]^ respiratory chain complex assembly,^[8]^ mitochondrial dynamics, and mitochondrial calcium dynamic balance.^[9]^

Pathogenic mutations of SCA28 are commonly reported to be heterozygous missense mutations within the key proteolytic domain encoded by exons 15 and 16. Other mutations include a missense mutation in exons 4^[10]^ and 10,^[5]^ a frameshift mutation in exon 15,^[11]^ and a deletion spanning exons 14 to 16.^[12]^Table 2 presents a literature review of pathogenic nucleotides. As shown, the glycine to arginine substitution caused by the c.2011G > C mutation is seen in the highly conserved peptidase-M41 region of the AFG3L2 protein.^[13]^ SCA28 was first reported 2 years after the discovery of mutations on chromosome 18p11.22-q11.2,^[14]^ showing that the disease-associated variants on chromosome 18p11.22-q11.2 span the genomic DNA region of 7.9 Mb, manifested as a G to A variant resulting in the substitution of glutamate and lysine residues.^[15]^ Concurrent heterozygote missense mutations (c.1847A > G [p.Y616C], c.2167G > A [p.V723M]) are rare, and decrease the levels of 2 mitochondrial proteins: TOMM70 (translocase of outer mitochondrial membrane 70) and respiratory chain complex V (ATPase), which causes mitochondrial structural defects and suppressed functions.^[16]^ However, no dominant ataxia, except SCA28, has been shown to be associated with mitochondrial dysfunction.

**Table 2:** Literature review for gene pathogenic nucleotides.

Author	Publication	Country	Site	Clinical manifestations
Cagnoli C^[20]^	2010	Italy	c.2011G>A	Not reported
Gorman^[21]^	2015	England	c.2011G>T	Progressive external ophthalmoplegia (PEO) and ptosis
Laszlo^[13]^	2017	Hungary	c.2011G>C	Ataxia, dysarthria and eye movement disorders
Cagnoli^[14]^	2006	Italy	18p11.22-q11.2	Juvenile-onset and slow progress; nystagmus, dysarthria, ataxia, tendon hyperreflexia, bilateral ankle clonus and Babinski sign
Ulf Edener^[22]^	2010	Germany	c.2098G>A	Early-onset slowly progressive cerebellar ataxia
SinemTunc^[16]^	2019	Germany	c.1847A>G, c.2167G>A	Early-onset slowly progressive cerebellar ataxia, bilateral ptosis and dysarthria

Although AFG3L2 gene variants are similar in variation type and position, the clinical phenotypes of SCA28 patients can be further modified by the severity of clinical symptoms and age of onset, including slow progressive gait abnormalities, ataxia, and oculomotor nerve abnormalities (such as ophthalmoplegia and ptosis).^[17]^ A large amount of research has shown that patients with SCA28 have normal cognitive function, while mild cognitive impairment or intelligence quotient decline is not common.^[18]^ In the present report, c.1852A > G in the exon region of the AFG3L2 gene was found in both the proband (III-3) and his cousin (III-5), resulting in a lysine-to-glutamate amino acid substitution (p.K618E). The clinical manifestations of the patients suggested SCA28, and such heterozygous mutations are not presented in any case report, which may provide a reference for future clinical diagnosis. The limitations of this report cannot be negated, the sick relatives of the last generation of the proband are not available for gene sequencing due to their death, and we failed to perform gene sequencing for more members of the family. To date, the pathogenic mutations reported here have not been reported. We need to further genotype non-diseased members of the family and normal healthy individuals for this mutation, and carry out further research using cell and animal experiments to determine whether the missense mutation is responsible for the pathogenesis of SCA28.

The clinical manifestations of SCA are diverse. Certain features are present in different SCA types, but the diagnosis of SCA must be referenced from molecular genetic examinations, rather than clinical manifestations or MRI presentations. There has been no specific treatment targeting SCA, while symptomatic treatment, such as drug treatment and rehabilitation treatment, is the mainstay but does not prevent disease progression. Gene therapy is potentially the most ideal treatment option,^[19]^ yet sufficient clinical evidence is needed to support its feasibility. Clinically, symptomatic treatment and achievement of symptom relief are the main strategies combating SCA, and genetic counseling and prenatal screening are encouraged for SCA patients and healthy people in their families. Further study of molecular biology and genetics may extend our understanding of SCA and help find more reliable methods for effective prevention and treatment.

## Acknowledgments

We gratefully acknowledge XL, JC for performing a research on the questions and for conducting the literature search. We thank all the study participants.

## Author contributions

LW analyzed and interpreted the patient data, and was a major contributor in JL and KC guided the writing. XL and JC helped to analyze and interpret the patient data. All authors read and approved the final manuscript.

**Conceptualization:** Xiaoyang Liu.

**Data curation:** Linlin Wang.

**Formal analysis:** Linlin Wang.

**Funding acquisition:** Jia Li.

**Investigation:** Linlin Wang.

**Methodology:** Chunyang Kang.

**Project administration:** Xiaoyang Liu.

**Resources:** Xiaoyang Liu.

**Software:** Chunyang Kang.

**Supervision:** Jiajun Chen.

**Validation:** Jiajun Chen.

**Visualization:** Jia Li.

**Writing – original draft:** Chunyang Kang.

**Writing – review & editing:** Jia Li.

## References

[1] Manto M Marmolino D. Cerebellar ataxias. Curr Opin Neurol 2009;22:419–29. 19421057 10.1097/WCO.0b013e32832b9897

[2] Coarelli G Brice A Durr A. Recent advances in understanding dominant spinocerebellar ataxias from clinical and genetic points of view. F1000 Res 2018;7:1781. 10.12688/f1000research.15788.1 PMC6234732 30473770

[3] Sun YM Lu C Wu ZY. Spinocerebellar ataxia: relationship between phenotype and genotype - a review. Clin Genet 2016;90:305–14. 27220866 10.1111/cge.12808

[4] Manto MU. The wide spectrum of spinocerebellar ataxias (SCAs). Cerebellum 2005;4:02–6. 10.1080/14734220510007914 15895552

[5] Di Bella D Lazzaro F Brusco A. Mutations in the mitochondrial protease gene AFG3L2 cause dominant hereditary ataxia SCA28. Nat Genet 2010;42:313–21. 20208537 10.1038/ng.544

[6] Quiros PM Langer T Lopez-Otin C. New roles for mitochondrial proteases in health, ageing and disease. Nat Rev Mol Cell Biol 2015;16:345–59. 25970558 10.1038/nrm3984

[7] Nolden M Ehses S Koppen M Bernacchia A Rugarli EI Langer T. The m-AAA protease defective in hereditary spastic paraplegia controls ribosome assembly in mitochondria. Cell 2005;123:277–89. 16239145 10.1016/j.cell.2005.08.003

[8] Atorino L Silvestri L Koppen M. Loss of m-AAA protease in mitochondria causes complex I deficiency and increased sensitivity to oxidative stress in hereditary spastic paraplegia. J Cell Biol 2003;163:777–87. 14623864 10.1083/jcb.200304112 PMC2173682

[9] Maltecca F Baseggio E Consolato F. Purkinje neuron Ca2+ influx reduction rescues ataxia in SCA28 model. J Clin Invest 2015;125:263–74. 25485680 10.1172/JCI74770 PMC4382234

[10] Qu J Wu CK Zuzuarregui JR Hohler AD. A novel AFG3L2 mutation in a Somalian patient with spinocerebellar ataxia type 28. J Neurol SciD 2015 530–1. 10.1016/j.jns.2015.10.003 26454370

[11] Musova Z Kaiserova M Kriegova E. A novel frameshift mutation in the AFG3L2 gene in a patient with spinocerebellar ataxia. Cerebellum 2014;13:331–7. 24272953 10.1007/s12311-013-0538-z

[12] Smets K Deconinck T Baets J. Partial deletion of AFG3L2 causing spinocerebellar ataxia type 28. Neurology 2014;82:2092–100. 24814845 10.1212/WNL.0000000000000491

[13] Szpisjak L Nemeth VL Szepfalusi N. Neurocognitive characterization of an SCA28 family caused by a novel AFG3L2 gene mutation. Cerebellum 2017;16:979–85. 28660440 10.1007/s12311-017-0870-9

[14] Cagnoli C Mariotti C Taroni F. SCA28, a novel form of autosomal dominant cerebellar ataxia on chromosome 18p11.22-q11. 2. Brain 2006;129:235–42. 16251216 10.1093/brain/awh651

[15] Mariotti C Brusco A Di Bella D. Spinocerebellar ataxia type 28: a novel autosomal dominant cerebellar ataxia characterized by slow progression and ophthalmoparesis. Cerebellum 2008;7:184–8. 18769991 10.1007/s12311-008-0053-9

[16] Tunc S Dulovic-Mahlow M Baumann H. Spinocerebellar ataxia type 28-phenotypic and molecular characterization of a family with heterozygous and compound-heterozygous mutations in AFG3L2. Cerebellum 2019;18:817–22. 31111429 10.1007/s12311-019-01036-2

[17] Adam MP Ardinger HH Pagon RA. Spinocerebellar Ataxia Type 28 – GeneReviews^®^. Handbook Clin Neurol 1993;103:461.

[18] Teive HAG Arruda WO. Cognitive dysfunction in spinocerebellar ataxias. Dement Neuropsychol 2009;3:180–7. 29213626 10.1590/S1980-57642009DN30300002 PMC5618971

[19] Miller VM Xia H Marrs GL. Allele-specific silencing of dominant disease genes. Proc Natl Acad Sci USA 2003;100:7195–200. 12782788 10.1073/pnas.1231012100 PMC165852

[20] Cagnoli C Stevanin G Brussino A. Missense mutations in the AFG3L2 proteolytic domain account for approximately 1.5% of European autosomal dominant cerebellar ataxias. Hum Mutat 2010;31:1117–24. 20725928 10.1002/humu.21342

[21] Gorman GS Pfeffer G Griffin H. Clonal expansion of secondary mitochondrial DNA deletions associated with spinocerebellar ataxia type 28. JAMA Neurol 2015;72:106–11. 25420100 10.1001/jamaneurol.2014.1753

[22] Edener U Wollner J Hehr U. Early onset and slow progression of SCA28, a rare dominant ataxia in a large four-generation family with a novel AFG3L2 mutation. Eur J Hum Genet 2010;18:965–8. 20354562 10.1038/ejhg.2010.40 PMC2987378

